# 90K predicts the prognosis of glioma patients and enhances tumor lysate-pulsed DC vaccine for immunotherapy of GBM *in vitro*

**DOI:** 10.18632/aging.202645

**Published:** 2021-03-03

**Authors:** Yu Zeng, Xin Chen

**Affiliations:** 1 Department of Neurosurgery, Xiangya Hospital, Central South University, Changsha 410008, Hunan Province, China; 2 National Clinical Research Center for Geriatric Disorders, Xiangya Hospital, Central South University, Changsha 410008, Hunan, China

**Keywords:** 90K, LGALS3BP, DC vaccine, immunotherapy, glioma

## Abstract

Objectives: This study aimed to investigate the relationship between 90K expression with glioma malignancy and prognosis. Additionally, the enhancement effect of 90K in the Dendritic cell (DC) vaccine for Immunotherapy of glioblastoma (GBM) was evaluated *in vitro*.

Methods: The expression of 90K protein in glioma tissues was detected by western blot. The relationship between the 90K expression and the tumor grade as well as the prognosis of patients was further analyzed by mining TCGA and CGGA database. The concentration of IL-12p70 and IL-10 was detected by ELISA. T lymphocyte proliferation and lethal effect of cytotoxic T cell (CTL) were detected by CCK-8.

Results: The expression of 90K was significantly higher in glioma than normal tissue and increased with tumor grade (P< 0.05). Higher 90K expression was observed in IDH wildtype glioma than IDH mutant and predicted worse overall survival for glioma patients. The concentration of IL-12p70 and IFN-γ was the highest in the Apoptosis U251-90K-DC group, in which group the ability to kill U251 cells by CTL was also the strongest.

Conclusion: 90K was a useful biomarker for glioma malignancy and patient prognosis. The appearance of 90K enhanced the effect of Apoptosis U251-DC vaccine for immunotherapy of GBM.

## INTRODUCTION

Glioma is the most common primary central nervous system tumor, accounting for 50% of the brain tumors. High-grade glioma like glioblastoma multiforme (GBM) is lethal and hard to treat with a median overall survival of 14-15 months despite surgery and combined radio- and chemotherapy [1]. Recently, mounting evidence showed the potentials of immunotherapy as a promising way for refractory glioma. Chimeric antigen receptor (CAR) T cell therapy, Oncolytic viruses, Immune-checkpoint inhibitors, and Dendritic cells (DC) vaccination were the most encouraging areas in glioma therapy [2, 3]. Dendritic cells are the most robust antigen-presenting cells. Evidence has shown DC-based vaccination could target and kill GBM cells efficiently with little damage to the surrounding healthy tissues [4–8]. It enhances the recognition of GBM cells by the patient's immune system, activates a broad, effective, and persistent immune response, and eliminates tumor cells. Liau et al showed a clinical phase I trial of DC vaccines from patients’ peripheral blood pulsed GBM antigen peptides successfully extended mean OS to 23.4 months [9]. Currently, the most widely used DC vaccine loading antigens include 1. DC vaccines that load tumor-specific or related antigens; 2. DCs loaded with tumor cell antigens; 3. transfecting tumor cell-associated antigen-encoding gene into DC [10–13]. However, these methods were either too inefficient or time-consuming to make the case. To improve the tumor-killing efficiency, a combination of tumor-specific antigen and apoptotic GBM tumor cell lysate was pulsed DC to improve the effect of immunotherapy.

90K, also known as galectin 3 binding protein, LGALS3BP, Mac-2-binding protein, has recently been identified as a tumor-associated antigen and a promising immunotherapy target. It was first found as a useful predictor marker of disease progression in human immunodeficiency virus infection [14, 15]. Subsequently, it was recognized as a novel circulating antigen with poor prognosis in breast cancer and non-Hodgkin’s-lymphoma [16, 17]. Overexpression of 90K correlated with tumor distant metastasis and advanced stage [18–20]. Nevertheless, it was also highly expressed in hepatocellular carcinoma [21], pleural mesothelioma [22], pancreatic carcinoma [23], non-small cell lung carcinoma [18], prostate cancer [24, 25], and colorectal cancer [26], but weakly expressed in tumor-free tissues [27]. Therefore, bearing testified-immunogenicity, 90K has been verified as a tumor-specific antigen of lung cancer [28]. Recently it was adopted to induce cytotoxic T lymphocytes for colon cancer Immunotherapy [29]. Furthermore, it was used to make the DC vaccine for colon cancer [30]. Therefore, we believe strategies based on 90K for immunotherapy are encouraging.

As a promising therapeutic target, the relationship between 90K expression and tumor malignancy in glioma remains largely unknown. Previous studies found 90K overexpressed in GBM cell lines, and engineered enhancement of 90K expression caused tumor growth inhibition by stimulation of the residual cell-mediated immune defense *in vivo* [27]. However, comprehensive reports of 90K expression in all gliomas are lacking so far. Therefore, we analyzed the protein expression of 90K in human glioma and tumor-free brain tissues, and further verified the relationship between 90K expression and glioma features using The Cancer Genome Atlas (TCGA) and Chinese Glioma Genome Atlas (CGGA) dataset. Also, we predicted the prognosis of glioma patients by the two datasets. Additionally, we analyzed the expression of 90K and DC-specific gene markers and found a positive correlation between them in TCGA and CGGA datasets. A novel DC vaccine pulsed by 90K and apoptotic U251 cells was further tested, which successfully improved the proliferation of CD4+T, CD8+T cells, and the secretion of IFN-γ. It also greatly enhanced the success rate of cytotoxic T lymphocytes (CTL) killing GBM cells. Collectively, these findings not only demonstrate that 90K is a meaningful biomarker for glioma malignancy and prognosis, but provide a novel strategy based on 90K-apoptotic tumor cell pulsed DC targeted vaccine for cancer immunotherapies.

## RESULTS

### 90K expression was significantly up-regulated in GBM

**Figure 1:**
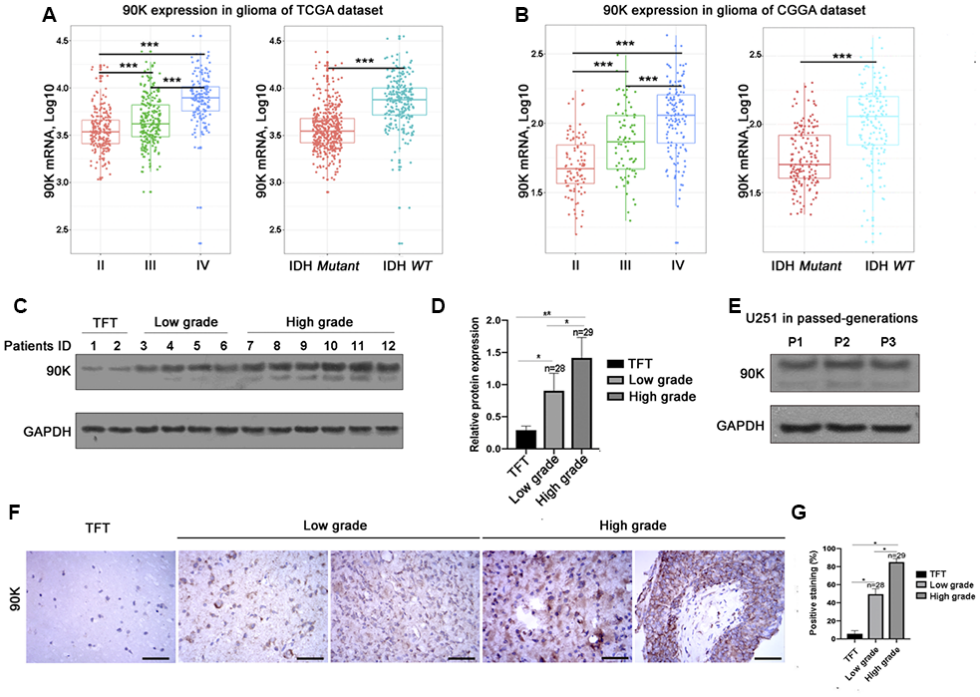
**Relationship between 90K expression and the clinical features of glioma.** The mRNA expression of 90K in glioma with different WHO grade and IDH status in the TCGA dataset (**A**) and CGGA dataset (**B**). Both datasets revealed 90K expression was higher in high grade glioma vs low grade; higher in IDH wildtype vs mutant (**A**, **B**). Protein expression pattern of 90K in glioma tissues, which showed 90K protein level was significantly increased in high grade glioma (**C**, **D**). Protein expression of 90K was stable in GBM cell line U251 by serial passaging. (**E**). Histological investigation via IHC staining also showed that 90K expression level significantly associated with pathological diagnosis and progression (**F**, **G**). * indicates p value < 0.05; ** indicates p value < 0.01; *** indicates p value < 0.001.

To analyze the expression of 90K in gliomas, we extracted clinical and genetic data from TCGA and CGGA datasets. First, we investigated 90K expression in gliomas based on WHO glioma grades. 90K expression was significantly up-regulated in higher grade gliomas. Specifically, WHO grade IV GBM demonstrated the highest expression, as compared to that in WHO grade II and grade III gliomas from the TCGA database (Figure 1A). Similar results were obtained using the CGGA dataset (Figure 1B). Moreover, a higher expression of 90K was observed in IDH wildtype glioma than that of IDH mutant in both datasets (Figure 1A, 1B). Second, the protein levels of 90K in 57 glioma samples were detected, lower 90K protein levels were found in tumor-free brain tissues. Moreover, its expression was gradually increased with tumor malignancy (Figure 1C, 1D and Supplementary Figure 2). Additionally, 90K protein expression in U251 cells was stably expressed by serial passaging (Figure 1E). Histological investigation via IHC staining also presented that 90K expression levels were significantly associated with pathological diagnosis and progression (Figure 1F, 1G). These results indicated that 90K was significantly enriched in glioma, particularly in GBM. Its expression was correlated with the malignancy of glioma, which could be potentially used as a target antigen for GBM immune therapy.

### 90K predicted worse survival in glioma patients and had a positive correlation with DC-specific marker genes

**Figure 2:**
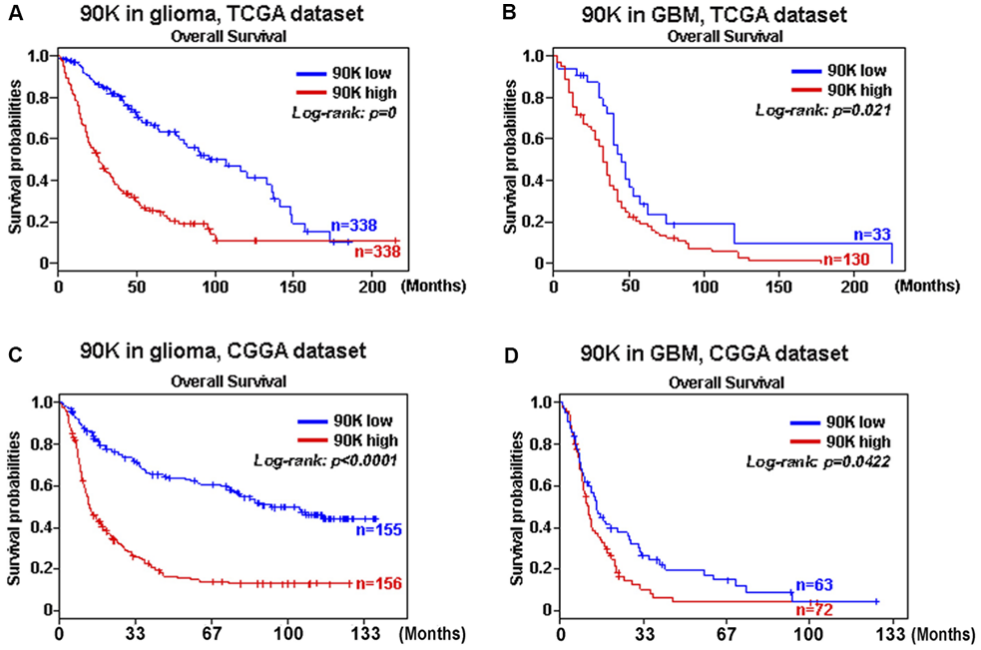
**The correlation between survival and 90K expression was analyzed.** Data showed that higher 90K expression was associated with worse overall survival (OS) in patients with glioma (**A**, **C**) and GBM (**B**, **D**) based on TCGA and CGGA datasets.

**Figure 3:**
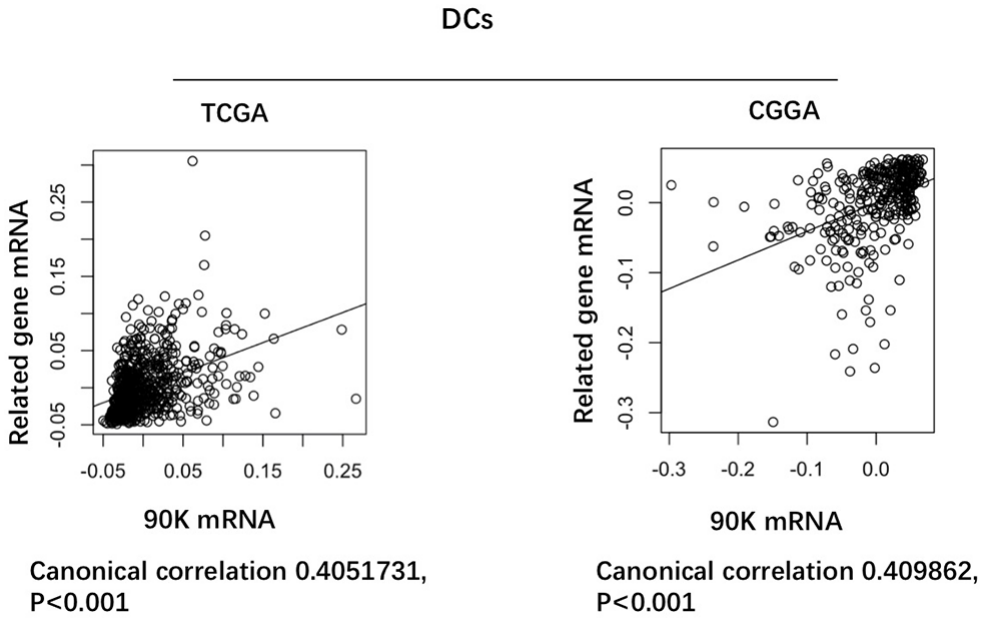
**Correlation of 90K expression with dendritic cell-specific marker genes in CGGA and TCGA datasets.** Each circle represents a patient with glioma.

Based on our findings that 90K was expressed at higher levels with higher grades of glioma, indicating a malignant biological property of this marker. Therefore, we evaluated the prognostic value of 90K based on TCGA and CGGA datasets to determine its effect on patient survival. Figure 2A depicts the Kaplan–Meier curve of the overall survival (OS) of patients with glioma. As shown in Figure 2A, 2B, a higher 90K expression predicted worse overall survival for glioma and GBM patients in TCGA dataset. Similarly, a strong association was observed between the higher expression of 90K and shorter patient OS for glioma and GBM patients in CGGA dataset (Figure 2C, 2D). Additionally, we analyzed the correlation of 90K expression with DC-specific marker genes in TCGA and CGGA, it showed 90K had a positive correlation with DC (Figure 3). These findings showed that 90K was a negative prognostic biomarker in glioma patients and could be used as an antigen for the DC vaccine.

### Heat shock was an efficient way to generate apoptotic U251 cells

**Figure 4:**
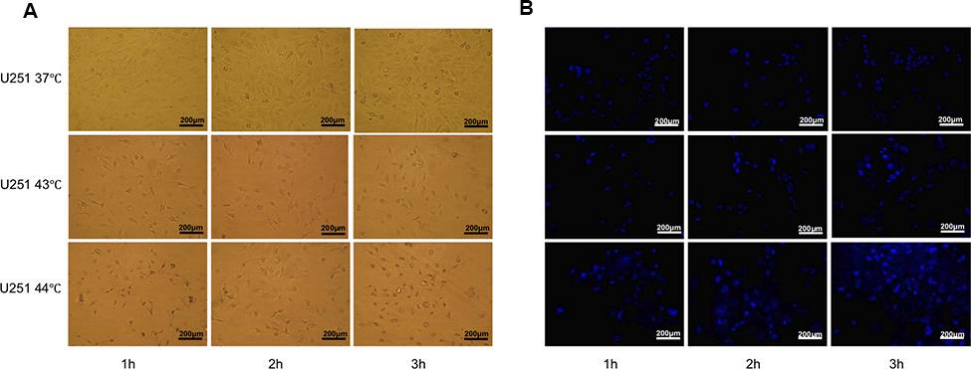
**Heat shock was an efficient way to generate apoptotic U251 cells.** (**A**) U251 cell morphology by heat induction 37° C, 43° C, 44° C for 1h, 2h, 3h respectively. With the prolongation of heat induction, the cells began to shrink, the cell density decreased, and the spacing between cells became significantly larger. (**B**) Apoptosis rate was calculated by Hoechst 33258 staining, which showed U251 cells had the highest apoptosis rate at 43° C 3h.

In order to make a robust DC vaccine, apoptotic U251 cells were attempted at 37° C, 43° C, 44° C for 1h, 2h, 3h respectively. With the prolongation of heat induction, U251 cell morphology began to change and the cells began to shrink. In addition, the cell density decreased, and the spacing between cells became significantly larger (Figure 4A). Then, the apoptosis rate was calculated by Hoechst 33258 staining. Few apoptoses of U251 cells in 37° C were detected in the staining, while in 43° C, 44° C group, apoptosis rate increased with the prolongation of heat, with the highest apoptosis in the 44° C group at 3h (Figure 4B). These results indicated heat shock was efficient to generate the apoptotic U251 cells for DC vaccines.

### Combination of 90K and apoptotic U251 cells improved the performance of DC vaccines

**Figure 5:**
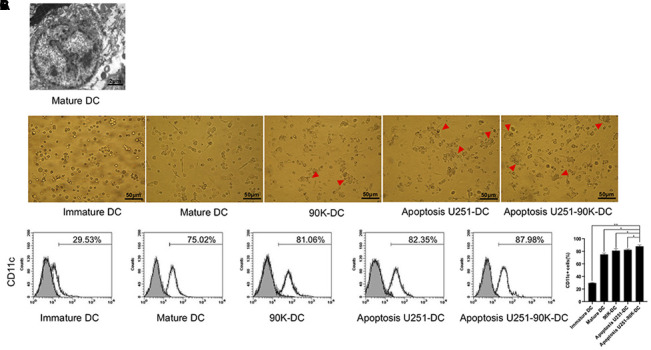
**Combination of 90K and apoptotic U251 cells improved the performance of DC vaccine.** (**A**) Mature DC under electric microscope. (**B**) Apoptosis U251-90K-DC group had more cells contact and fusion than other groups (red arrowhead). (**C**) The expression of CD11c on immature DC, Mature DC, 90K-DC, Apoptosis U251-DC and Apoptosis U251-90K-DC groups.

Typical DC characteristics were examined under the transmission electron microscopy, and the features of mature DC were as following: many dendritic protrusions and folds around the cells, the heterochromatin in the nucleus was highly condensed along the edge of the nucleus, the mitochondria in the cells were abundant, and multivesicular bodies were observed (Figure 5A). Under the microscope, there were few protrusions on the surface of the immature DC cells on day 6 after culture. However, on day 8, it showed a lot of burr-like protrusions on the mature DC cell surface. When 90K or apoptotic U251 cells were co-cultured with immature DC, cells were in close contact and some were fused on day 8, obvious fusion was observed in the Apoptosis U251-90K-DC group (Figure 5B). In addition, the expression of CD11c on the surface of DC cells was detected in each group. The percentage was 29.53±0.65 %, 75.02±2.19 %, 81.06±3.17 %, 82.35±1.28 %, 87.98±2.10% in Immature DC, Mature DC, 90K-DC, Apoptosis U251-DC, Apoptosis U251-90K-DC respectively. Notably, the expression of CD11c in the Apoptosis U251-90K-DC group was higher than in other groups (Figure 5C). These findings demonstrated that the combination of 90K and apoptotic U251 cells improved the performance of DC vaccines than using apoptotic U251 cells or 90K alone.

### Combination of 90K and apoptotic U251 cells-pulsed DC vaccine increased the proliferation effect of initial T lymphocytes and altered secretion of related interleukins

**Figure 6:**
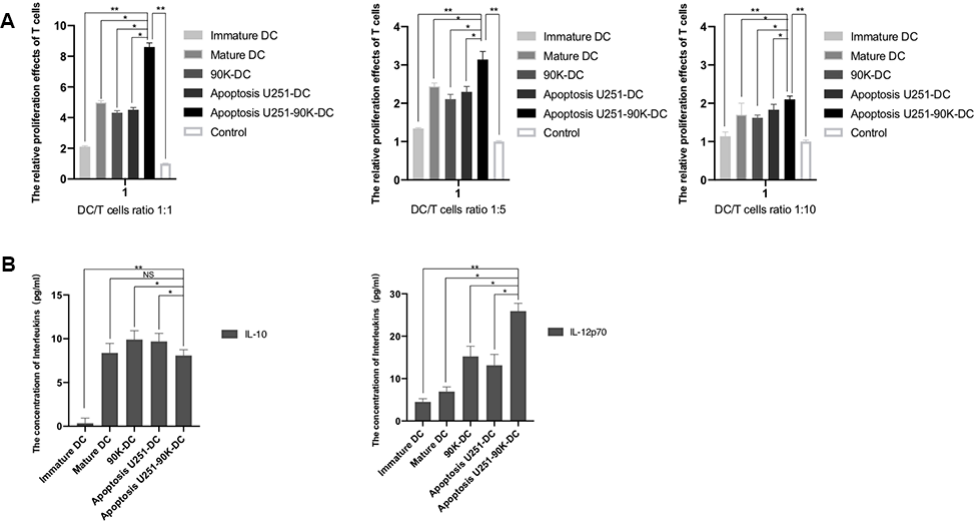
**Combination of 90K and apoptotic U251 cells-pulsed DC vaccine increased the proliferation effect of initial T lymphocytes and altered secretion of related Interleukins.** (**A**) The relative proliferation effects of initial T lymphocytes induced by immature DC, Mature DC, 90K-DC, Apoptosis U251-DC and Apoptosis U251-90K-DC (n=5). (**B**) The secretion of IL-10 and IL-12p70 induced by the above groups (n=5). * indicates p value < 0.05; ** indicates p value < 0.01.

To evaluate the effects of stimulation of initial T cells stimulated by different DC vaccines, T cells from PMBC were selected. The positive rate of CD4+ T cells after sorting was 98.18±0.76% vs 29.81±1.65% prior sorting, and CD8+ T cells after sorting were 98.40±0.42%vs 21.79±1.02% prior sorting. Then, The antigen-loaded DCs of each group were co-cultured with the initial T lymphocytes in different DC/T lymphocytes ratio as 1:1, 1:5, and 1:10. As showed in Figure 6A, the Apoptosis U251-90K-DC group had more T lymphocyte proliferation than other groups, extraordinarily in the Apoptosis U251-90K-DC group with ratio1:1. Additionally, secretion of immune inhibitor IL-10 and immune stimulator IL-12p70 by different DC vaccines were tested. As confirmed in Figure 6B, IL-10 was little detected in the Immature DC group and decreased in Apoptosis U251-90K-DC group compared to 90K-DC or Apoptosis U251-DC groups, while IL-12p70 was the lowest in the Immature DC group and highest in Apoptosis U251-90K-DC group. These results indicated that the combination of 90K and apoptotic U251 cells-pulsed DC vaccine increased the proliferation of T lymphocytes and altered secretion of IL-10 and IL-12p70, which could help to target immune kill of GBM.

### Combination of 90K and apoptotic U251 cells-pulsed DC vaccine increased the IFN-γ secretion of CD4+T and CTL cells and the GBM lethal effect of CTL cells

**Figure 7:**
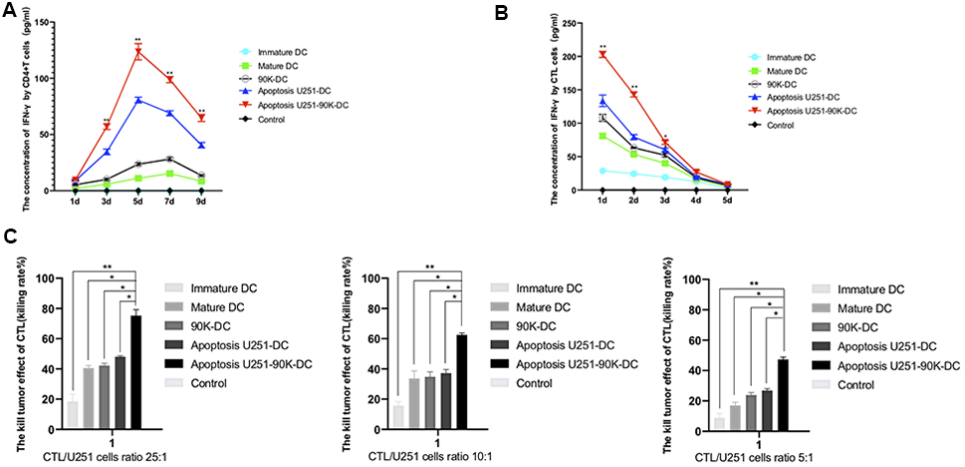
**Combination of apoptosis U251 cells and 90K protein pulsed DC vaccine increased the IFN-γ secretion of CD4+T and CTL cells and the GBM lethal effect of CTL cells.** (**A**) The secretion of IFN-γ in CD4+T cells by different DC vaccines (n=5). (**B**) The secretion of IFN-γ in CTL cells by different DC vaccines (n=5). (**C**) The lethal effect of CTL to U251 cells by co-cultured with different DC vaccines by CTL/U251 cells ratio at 25:1, 10:1, 5:1 (n=5). * indicates p value < 0.05; ** indicates p value < 0.01.

To assess the practical killing effects of different DC vaccines, IFN-γ secretion of CD4+T and CTL cells and GBM lethal effects of CTL were tested. As showed in Figure 7A, the concentrations of IFN-γ of CD4+ T cells increased from day 1 to day 5 and then decreased in Apoptosis U251-DC, Apoptosis U251-90K-DC, 90K-DC, Mature DC, and Immature DC groups, Apoptosis U251-90K-DC had more secretion of IFN-γ than other groups. The concentrations of IFN-γ of CTL cells decreased from day 1 to day 5 in the above groups, also Apoptosis U251-90K-DC had more secretion of IFN-γ than other groups (Figure 7B). These results indicated that CD4+ T and CTL cells secreted more IFN-γ under the stimulation of 90K protein. Furthermore, the lethal effect of CTL to U251 cells by co-cultured with different DC vaccines, the ratio of CTL/U251 cells was 25:1, 10:1, 5:1. As indicated in Figure 7C, The lethal effect increased with the ratio of CTL/U251 cells increased, simultaneously, the Apoptosis U251-90K-DC group had the best lethal effect on U251 cells with different ratios. Taken together, 90K enhanced the effect of apoptotic U251 cells-pulsed DC vaccine to induce CTL to kill GBM cells.

## DISCUSSION

GBM is the most common malignant primary brain tumor. The standard management of newly diagnosed GBM includes surgery followed by concurrent radiotherapy with temozolomide and further adjuvant temozolomide. Despite advances in the therapeutic approach, the median overall survival of GBM is 14-15 months. Consequently, there is a clear need for improved therapeutic strategies. Tumor treating fields (TT fields), target therapy, CAR-T cell therapy, Oncolytic viruses, immune-checkpoint blockade (ICB), novel drugs, and vaccines were the most encouraging areas in glioma therapy. Combinations of treatment especially in brain tumors will be a trend since Ionizing radiation (IR) can activate the immune response and ICB can alleviate the immune-repression tumor microenvironment [31–34]. Another rational combination approach is to use ICB and vaccines, given their potential to both generate new antigen-specific T cell responses against tumor cells and amplify existing responses [35]. As one of the most promising immunotherapy approaches, Dendritic cells (DCs) are broadly used as vaccines in different types of cancers, such as lung cancer, melanoma, gastric cancer, prostate cancer, breast cancer, ovarian cancer, lymphoma, and GBM [6–8, 35–41]. A combination of DC vaccination with ICB may be one of the best solutions to improve the efficacy of ICB to treat GBM with low mutational burdens [42]. Discovering the effective and efficient antigen to pulse DC is of great importance. In this study, tumor-associated antigen 90K combination with apoptotic U251 cells greatly helped to make DC vaccines and kill GBM cells.

90K is a member of the Scavenger Receptor Cysteine-Rich family, which also including CD5, CD6, M130, butiyinzi1, and other immune defense and immune control proteins [43]. The expression of 90K is low in the brain, bone marrow, liver, and kidney, while high in lung, colon, and stomach, and extremely high in HIV, autoimmune diseases, and cancers [14, 18, 27, 44]. It’s a diagnostic and prognostic biomarker in many malignant tumors including breast cancer, lung cancer Non-Hodgkin’s Lymphomas, and colon cancer [14, 16, 18–20, 26, 28]. Similarly, we found 90K predicts poor prognoses in gliomas. The expression of 90K was higher in IDH wild type glioma, which may work with IDH to predict the prognosis of glioma patients. In this study, we also found the protein expression of 90K is very low in tumor-free brain tissues, which is consistent with Jallal B’s report who detected low 90K mRNA expression by PCR [27]. The expression of 90K in glioma is much higher than the brain, and correlated with glioma malignancy, eminently increased in GBM, which indicates that it is a glioma-related antigen, and could be used as an immune therapy target in GBM. 90K was not only a tumor-related antigen but an immune stimulator. Ullrich et al. [18] found that 90K could stimulate the secretion of IL-2, which could activate natural killer cell and lymphokine-activated killer. Natoli et al. [45] showed that 90K greatly promoted immune reaction in breast cancer by inducing IL-2 and MHC I. In our study, 90K was also found to have a close correlation with DC-specific marker genes, which may be used as an antigen for the DC vaccine. Taken together, 90K is a good target for GBM immunotherapy.

To better understand the effect and mechanism of 90K in DC vaccines, different groups of DC vaccines were made including Immature DC, Mature DC, 90K-DC, Apoptosis U251-DC, Apoptosis U251-90K-DC. Then, IL-10, IL-12p70, and IFN-γ of these groups were examined, we found that IL-12p70 and IFN-γ were the highest in the Apoptosis U251-90K-DC group while IL-10 of that group was lower than Mature DC, 90K-DC, and Apoptosis U251-DC groups. IL-12p70 can promote the differentiation of CD4+ T lymphocytes into Th1, while IL-10 can inhibit the differentiation of CD4+ T lymphocytes into Th1. Therefore, it demonstrated that the 90K enhanced apoptotic U251 cells-loaded DC effectively, inducing the differentiation of CD4+ T lymphocytes into Th1, which helped to activate the GBM-associated antigen-specific immune response-dependent pathway. The cytokines secreted by Th1 mainly included IL-2, IL-12, IFN-γ, and TNF-β. IL-2 and IFN-γ can promote CTL by up-regulating the expression of MHC class I, II molecules, and adhesion molecules [46]. Activated CTL can produce a large amount of IFN-γ, which could also induce DC secretion of IL-12, which shows the cross-activation between the three achieves efficient activation of CTL [47]. In the present research, we detected the concentrations of IFN-γ secreted by CD4+ T cells and CTL and found that the concentration of IFN-γ in Apoptosis U251-DC, 90K-DC, and Apoptosis U251-90K-DC groups was significantly higher than that of non-antigen-loaded DC (Immature DC and Mature DC group). Moreover, the concentration of IFN-γ secreted by Apoptosis U251-90K-DC group was even higher than 90K or apoptosis U251 alone. These findings indicated that 90K enhanced the effect of apoptotic U251 cells-pulsed DC to induce a more intense GBM-associated antigen-specific immune response, which could improve the effectiveness of the DC vaccine in killing GBM cells. To further verify the hypothesis, GBM U251 cells were co-cultured with these DC vaccines, it showed that the CTL activated by Apoptosis U251-90K-DC group had the highest killing rate of U251 cells than other groups.

In summary, these findings demonstrated 90K was a useful predictor for glioma malignancy and prognosis. Moreover, 90K played an important role in immunotherapy against GBM and could significantly enhance the anti-GBM effects of DC vaccines.

## MATERIALS AND METHODS

### Patients and clinical samples

A total of 57 adult glioma specimens. These specimens were obtained from patients who underwent either explorative or radical surgery at the Xiangya Hospital of Central South University (Hunan, China) after informed consent was obtained. Historical diagnosis of glioma was performed according to WHO classification, including 28 tumor specimens of low-grade diffuse glioma, 11 tumor specimens of glioma grade III, and 18 tumor specimens of GBM. Demographic information was organized in Table 1. For comparisons, two tumor-free brain tissue samples were obtained from patients who were injured in traffic accidents and underwent a decompressive operation. The clinical and RNA-seq data from 311 glioma patients in the CGGA dataset, ranging from WHO grade II to grade IV, were included in the analysis. (http://www.cgga.org.cn). To maintain consistency, we also validated our findings using the TCGA dataset, which including clinicopathological characteristics and RNA-seq data from 676 glioma samples. (http://cancergenome.nih.gov).

**Table 1:** Patient demographic information.

**Clinical Characteristics**	**N (%)**
Age, years, mean (range)	34.9 (19-70)
Sex	
Male	30 (52.6)
Female	27 (47.4)
Surgery	
Total	42 (73.7)
Subtotal	15 (26.3)
WHO grade and IDH status	
II Wild-type	6 (10.5)
II Mutant	22 (38.6)
III Wild-type	3 (5.3)
III Mutant	8 (14)
IV Wild-type	16 (28.1)
IV Mutant	2 (3.5)
MGMT methylation	
Methylated	34 (59.6)
unmethylated	23 (40.4)

### Cell culture and heat shock U251 cells

The human malignant GBM U251 cell line was purchased from The American Type Culture Collection (ATCC). Cells were cultured in RPMI media 1640 supplemented with 10% FBS and penicillin G (100 U/mL)/streptomycin (100 μg/mL), at 37° C containing 5% CO_2_. The well-grown U251 cells were seeded in T75 treated flask, tips were sealed and placed in 43° C or 44° C water bath for 1 h, 2 h, or 3 h, and then changed with fresh medium back in incubators for 12 hours. Cells were observed under microscopes and compared with the normal 37° C U251 cells.

### Hoechst 33258 staining

The cells were fixed with 2–4% formaldehyde for 10 minutes, then immersed in PBS for 10 minutes, stained with Hoechst 33258 for 5 minutes, and then mounted. Cells were observed and counted under a fluorescence microscope. The apoptosis rate was counted in 5 fields.

### DC collection, culture, characterization, and pulsed with antigen

The workflow was demonstrated in Supplementary Figure 1. Briefly, take 10 ml of fresh anticoagulant blood mixed with 10 ml D'hanks solution, add 10 ml of cell separation solution, and centrifuge at 2000 rpm for 20 minutes. The second layer of cells was carefully collected and mixed with 5 times’ volume of D'hanks solution and centrifuge at 1500 rpm for 15 minutes. Repeated twice to get the lymph cells. Seeded cells at 2 × 10^6^ cells/mL into 6 well plate, transferred the supernatant to another well after standing 4 hours, left adherent cells were pre-DC cells, washing with 2ml PBS, then, add 1000U/ml rhGM-CSF, 1000U/ml rhIL-4 with RPMI 1640 media, half media change every two days. Then, added rhTNF-α or 90K or apoptotic U251 cells for 48 hrs (Supplementary Figure 1). 90K peptide was purchased from Sigma-Aldrich (CAT# APREST76118) and used at a concentration of 1 μg/ml. Then, cells were observed under electron microscopy and detected CD11c by flow cytometry.

### Immunophenotyping

CD11c fluorescein isothiocyanate (FITC) (BD Biosciences, CAT#561355) was used to analyze for immunophenotyping, and the samples were analyzed using a BD fluorescence cytometer and CellQuest software within 1 h.

### Isolation of CD4+T and CD8+T cells by immunomagnetic beads

CD4+T and CD8+T cells were isolated by human CD4+ T Cell Isolation Kit or human CD8+ T Cell Isolation Kit following the protocol from Miltenyi biotec using MidiMACS.

### CCK-8 measurement

The cell counting kit-8 (CCK8) (C0037, Beyotime, China) was used according to the manufacturer’s instructions. DC cells were co-cultured with T cells, the single cultured T cells were the normal control group, the cell number ratio of DC/T cell was: 1:1, 1:5, 1:10 for initial T cell proliferation rate. While for U251 kill rate detection, the cell number ratio of CTL/U251 was: 25:1, 10:1, 5:1. Briefly. T cells were placed into 96-well plates (2×10^3^cells/well), add IL-2 200u/ml, 5 days later, CCK-8 solution (approximately 10 μl per well) was added, and cultivated for 1 h under the same conditions. After vertexing for 60s and suspension, the absorbance at 450 nm was measured. The dye consisted of a 2-(2-methoxy-4-nitrophenyl)-3-(4-nitrophenyl)-5-(2,4-disulfophenyl)-2H-tetrazolium monosodium salt (WST-8) reduction by NADH from living cells. Each experiment was repeated 3 times.

### Elisa

IL-10 (BioLegend, #430607), IL-12p70 (Cell Sciences, #CKH154), and IFN-γ (BioLegend, #430107) in the supernatant of each group were analyzed using ELISA kits. Proteins were quantified with ELX-800 Universal Microplate Reader (Bio-Tek, Winooski, VT, USA) using Gen5™ software (Bio-Tek, Instruments, VT, USA).

### Western blot

Tumor tissues or cells were solubilized in cold RIPA lysis buffer and then separated with 10% SDS-PAGE. After SDS-PAGE, proteins were transferred from the gel to a PVDF membrane. Membranes were blocked in 5% non-fat dried milk in PBST for 2 h and then incubated overnight with anti-90K (1:800), anti-GAPDH (1:1000) antibodies obtained from Santa Cruz Biotechnology, Inc. (Santa Cruz #374541, #365062). After incubation with the appropriate secondary antibody, immune complexes were detected using an ECL kit. Results were visualized by autoradiography using preflashed Kodak XAR film.

### Immunohistochemical staining

Briefly, five micron thick sections were deparaffinized with xylene, followed by gradation washes in 100%, 95%, 80%,70% ethanol before performing heat antigen retrieval in 10 mM Sodium Citrate buffer (pH 6.0) for 20 minutes. Subsequently, sections were treated with peroxidase block (Dako) for ten minutes followed by overnight incubation at 4° C with the following primary antibodies: anti-90K 1:400 (Santa Cruz #374541), After multiple washes with 1X Tris-buffered saline and Tween 20 (TBST) solution, slides were incubated at room temperature for two hours with corresponding species-specific horseradish peroxidase (HRP) conjugated secondary antibody from EnVision+ System (Dako, K500711-2). Signal was visualized by the HRP-DAB reaction. Counterstaining for nuclei was performed using Mayer’s hematoxylin stain followed by graded dehydration and xylene washes. Coverslips were mounted with Permount (Fisher Scientific). Antibody validation is provided on the manufacturers’ website. Diaminobenzidine (DAB), brightfield staining was performed according to standard protocols using DAB EnVision+ System (Dako) on paraffin sections. The images of immunohistochemical staining were acquired by an Olympus microscope.

### Statistical analyses

Values were expressed as the mean ± standard deviations of the mean. Statistical analysis was performed using Student’s t-test or one-way analysis of variance. Statistical analysis was performed using SPSS 18.0 software (SPSS, Inc., Chicago, IL, USA). The prognostic value of 90K was investigated by Kaplan–Meier analysis using R language (survival package) [48]. P ≤ 0.05 was considered to indicate a statistically significant difference.

### Availability of data and materials

The datasets generated and analyzed during the current study are available from the corresponding author on reasonable request.

### Ethical approval and ethical standards

The study with primary human cells was approved by the ethics committee of the Xiangya Hospital, Central South University (No. 2016121015) and the procedures with human samples were performed in accordance with ethical standards of the ethics committee and the Helsinki Declaration of 1975 and its later amendments.

## Supplementary Material

Supplementary Figures
