# Supplementary figures and images for: 90K predicts the prognosis of glioma patients and enhances tumor lysate-pulsed DC vaccine for immunotherapy of GBM in vitro
Source: Aging (Albany NY). 2021 Mar 3;13(6):8355–68. doi: 10.18632/aging.202645 (PMC8034892; doi:10.18632/aging.202645)

[www.aging-us.com](http://www.aging-us.com)

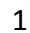

## AGING

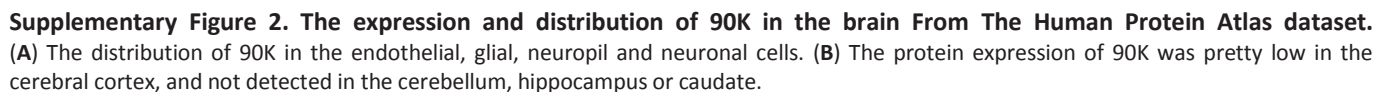

Supplement: Supplementary Figures [file aging-13-202645-s001.pdf]
